# KIF3A accelerates KIF3C within the kinesin-2 heterodimer to generate symmetrical phosphate release rates for each processive step

**DOI:** 10.1074/jbc.RA120.015272

**Published:** 2020-11-22

**Authors:** Sean M. Quinn, Troy Vargason, Nilisha Pokhrel, Edwin Antony, Juergen Hahn, Susan P. Gilbert

**Affiliations:** 1 Department of Biological Sciences, Rensselaer Polytechnic Institute, Troy, New York, USA; 2 Center for Biotechnology and Interdisciplinary Studies, Rensselaer Polytechnic Institute, Troy, New York, USA; 3 Department of Biomedical Engineering, Rensselaer Polytechnic Institute, Troy, New York, USA; 4 The Department of Biology, Marquette University, Milwaukee, Wisconsin, USA; 5 The Department of Biochemistry and Molecular Biology, Saint Louis University School of Medicine, St Louis, Missouri, USA; 6 Department of Chemical & Biological Engineering, Rensselaer Polytechnic Institute, Troy, New York, USA

**Keywords:** ATPase, fluorescence, intracellular transport, kinesin, mathematical modeling, microtubule, molecular motor, neuron, presteady-state kinetics, KOH, potassium hydroxide, MDCC, *N*-[2-(1-maleimidyl)ethyl]-7-(diethylamino) coumarin-3-carboxamide, MgATP, magnesium ATP, MSE, mean-squared error, MT, microtubule, MW, molecular weight, NaCl, sodium chloride, ODE, ordinary differential equation, PBP, phosphate-binding protein, PNPase, purine nucleoside phosphorylase, TEV, tobacco etch virus

## Abstract

Heterodimeric KIF3AC is a mammalian kinesin-2 that is highly expressed in the central nervous system and associated with vesicles in neurons. KIF3AC is an intriguing member of the kinesin-2 family because the intrinsic kinetics of KIF3A and KIF3C when expressed as homodimers and analyzed *in vitro* are distinctively different from each other. For example, the single-molecule velocities of the engineered homodimers KIF3AA and KIF3CC are 293 and 7.5 nm/s, respectively, whereas KIF3AC has a velocity of 186 nm/s. These results led us to hypothesize that heterodimerization alters the intrinsic catalytic properties of the two heads, and an earlier computational analysis predicted that processive steps would alternate between a fast step for KIF3A followed by a slow step for KIF3C resulting in asymmetric stepping. To test this hypothesis directly, we measured the presteady-state kinetics of phosphate release for KIF3AC, KIF3AA, and KIF3CC followed by computational modeling of the KIF3AC phosphate release transients. The results reveal that KIF3A and KIF3C retain their intrinsic ATP-binding and hydrolysis kinetics. Yet within KIF3AC, KIF3A activates the rate of phosphate release for KIF3C such that the coupled steps of phosphate release and dissociation from the microtubule become more similar for KIF3A and KIF3C. These coupled steps are the rate-limiting transition for the ATPase cycle suggesting that within KIF3AC, the stepping kinetics are similar for each head during the processive run. Future work will be directed to define how these properties enable KIF3AC to achieve its physiological functions.

Heterodimeric KIF3AC is a mammalian kinesin-2 that is highly expressed in brain and the spinal cord, yet its physiological function and intracellular transport properties are not well understood (reviewed in Refs. (1, 2, 3, 4)). We know very little about the adaptors for cargo linkage or the cargo that KIF3AC transports although recent reports have provided some insight (5, 6). The mammalian kinesin-2 motors result from four genes, kif3a, kif3b, kif3c, and kif17 (7, 8, 9, 10, 11, 12). Although KIF17 exists as a homodimer (12), expression of kif3a, kif3b, and kif3c can result in heterodimeric KIF3AC and KIF3AB motors (8, 9, 13). However, note that KIF3A and KIF3B do not form homodimers, and KIF3C does not form a heterodimer with KIF3B (9, 11, 13, 14). Moreover, KIF3C is primarily expressed in neurons (11). These studies suggest that KIF3AB, KIF3AC, and KIF17 may have distinct functions in neurons.

Our single-molecule quantum dot motility assays revealed that in the absence of load, KIF3AB, KIF3AA, and KIF3BB were quite similar in velocity with KIF3AB at 246, KIF3AA at 293, and KIF3BB at 328 nm/s (4, 15). In addition, their run lengths were determined with KIF3AB at 1.62, KIF3AA at 0.98, and KIF3BB at 1.51 μm. Moreover, their single-molecule stepping properties under load were also similar suggesting that KIF3AB behaves more similar to a homodimer than a heterodimer (16, 17). However, KIF3AC has remained a conundrum because of the differences in the mechanochemical capability of the KIF3A motor domain in comparison to KIF3C. The challenge has been to define the transport potential of the KIF3AC heterodimer whose individual polypeptides appear so different.

The single-molecule motility assays revealed that in the absence of load, KIF3AC was highly processive with a run length of 1.23 μm and velocity at 186 nm/s (Refs. (4, 15); Table 1). In contrast, the engineered KIF3AA and KIF3CC homodimers were very different from each other with the KIF3AA run length at 0.98 μm and velocity at 293 nm/s in comparison to KIF3CC whose run length was decreased to 0.57 μm with an exceedingly slow velocity at 7.5 nm/s (Table 1). Moreover, Deeb *et al*. (18) recently reported that it is the KIF3A motor domain that enables KIF3AC to switch microtubule (MT) tracks at MT intersections, whereas KIF3CC mainly detached at intersections (67.7%). Therefore, there are clear mechanochemical differences in KIF3A in comparison to KIF3C in spite of the high sequence similarity in their motor domains and neck linkers (4, 19).

**Table 1:** Experimentally determined KIF3 constants (4, 15, 20, 21)

Kinetic step	Constant	Units	KIF3AC	KIF3AA	KIF3CC
MT association	*k*_+1_	μM^−1^s^−1^	6.6 ± 0.2	11.4 ± 0.6	2.1 ± 0.1
	*k*_−1_	s^−1^	—	2.4 ± 0.9	0.6 ± 0.1
ADP release	*k*_+2_	s^−1^	42.5 ± 0.9^a^	77.7 ± 1.4	7.6 ± 0.1
	*K*_1/2,MT_	μM	7.5 ± 0.4^a^	4.4 ± 0.2	1.7 ± 0.1
MantATP binding	*k*_+3_	μM^−1^s^−1^	11.0 ± 0.6	16.0 ± 0.5	0.68 ± 0.04
	*k*_−3_	s^−1^	21.4 ± 7.2	10.5 ± 5.1	7.7 ± 0.4
ATP isomerization	*k*_+4_	s^−1^	81.0 ± 1.0	ND	ND
ATP hydrolysis	*k*_+5_	s^−1^	69.1 ± 1.2	ND	ND
	*A*_max_		0.77 ± 0.02 per site	ND	ND
P_*i*_ release^a^	*k*_+6_	s^−1^	42.6 ± 0.6	45.1 ± 1.7	12.1 ± 0.9
	*K*_1/2,ATP_	μM	28.2 ± 1.8	30.9 ± 4.7	13.9 ± 3.5
Steady state	*k*_cat_	s^−1^	21.5 ± 0.3	34.7 ± 0.5	1.1 ± 0.02
	*K*_*m*,ATP_	μM	138.1 ± 12.8	47.7 ± 0.1	4.8 ± 0.5
	*K*_1/2,MT_	μM	0.23 ± 0.03	0.19 ± 0.002	0.04 ± 0.007
Single-molecule velocity	—	nm/s	186.5 ± 5.6	293.2 ± 4.2	7.5 ± 0.4

MantATP, 2'-(or-3')-*O*-(*N*-Methylanthraniloyl) Adenosine 5'-Triphosphate; MT, microtubule; ND, not determined; — not observed.

a KIF3 constants determined from the experimental data presented in this study.

Our previous experimental work of presteady-state kinetics focused on entry into the processive run and showed that the MT association rate constant for KIF3AC at 6.6 μM^−1^s^−1^ was less than that of KIF3AA at 11.4 μM^−1^s^−1^ but significantly faster than KIF3CC at 2.1 μM^−1^s^−1^ ((20); Table 1). The computational modeling revealed that although there was an equal probability of either KIF3A or KIF3C initiating the processive run (Fig. 1), the results predicted that the rate for MT association for KIF3A and KIF3C became similar at 3.3 μM^−1^s^−1^ (21). This result was surprising and suggested that the rate of MT association was due to heterodimerization. In contrast, the model predicted that the subsequent steps of ADP release after MT collision followed by ATP binding were consistent with the intrinsic kinetics of KIF3AA or KIF3CC. For the previous computational work, the latter steps in the ATPase cycle including ATP hydrolysis followed by phosphate release coupled to motor head detachment, and ATP binding to the new leading head was collapsed into a single first-order rate constant (Fig. 1*A*; E5A–E2C and E5C–E2A). When modeled, the results revealed fast kinetics for KIF3A at 90 s^−1^ and 13 s^−1^ for KIF3C suggesting that KIF3A would undergo a fast step and KIF3C would undergo a significantly slower step during a processive run (21). However, the earlier computational modeling did not include experimental data to evaluate phosphate release coupled with motor detachment from the MT. This transition has been thought to be the rate-limiting step of the ATPase cycle (20) and therefore, a better gauge of step duration.

**Figure 1:**
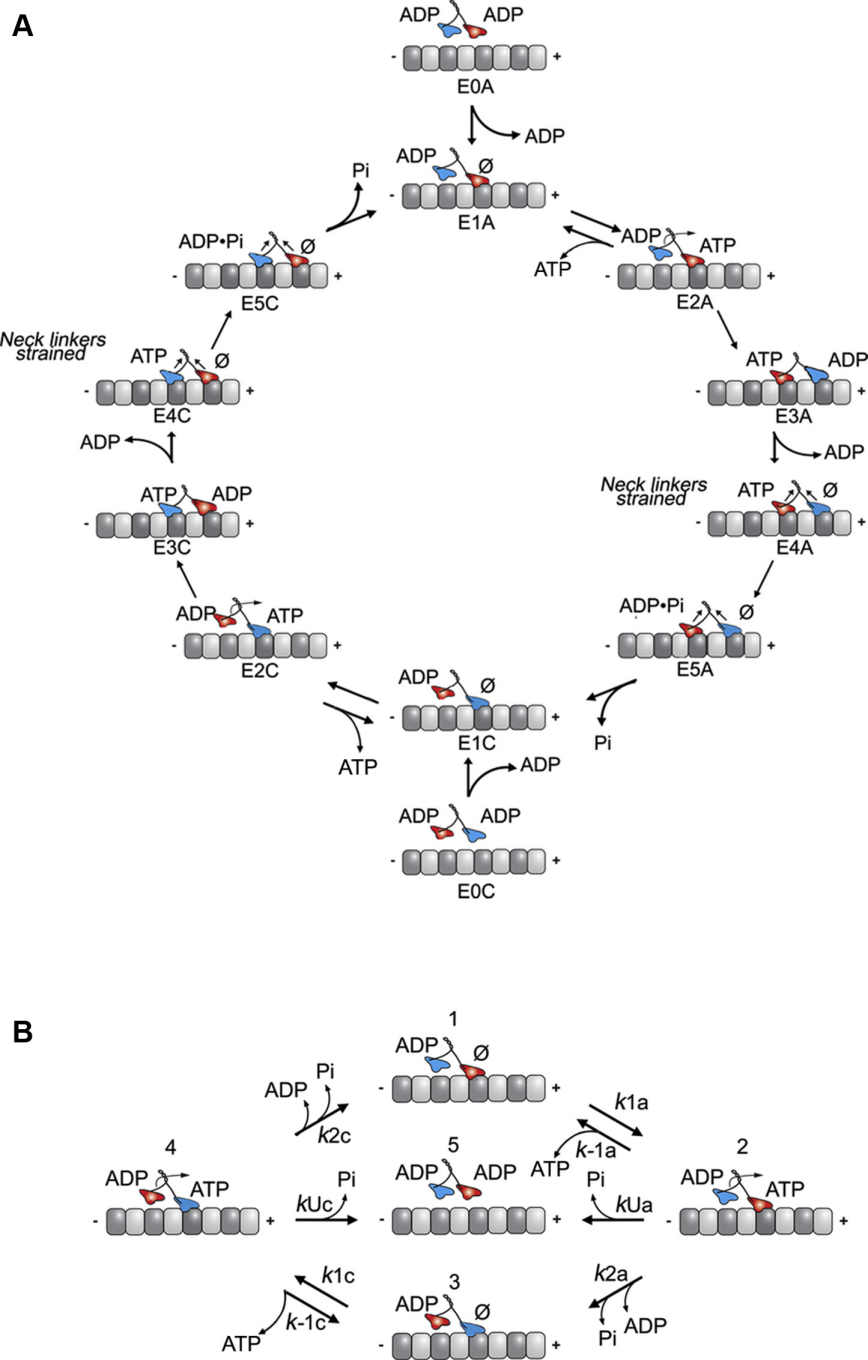
**KIF3AC stepping cycle and computational scheme.***A,* the cycle begins when one motor domain associates with the MT (E0A–E1A or E0C–E1C). Note that the KIF3A head is colored *red* and KIF3C *blue*. On MT association, ADP is released from the MT-interacting head, and ATP binds to the vacant nucleotide-binding site (E0A–E2A or E0C–E2C). Neck-linker docking results in a series of structural transitions where the tethered trailing head moves forward 16 nm, associates with the MT, and releases ADP (E2A–E4A or E2C–E4C). The ATP on the rear head is subsequently hydrolyzed, which is followed by coupled phosphate release from the active site and motor head dissociation (E4A–E1C or E4C–E1A). *B,* the computational scheme consists of five different intermediates. Either KIF3A or KIF3C motor head associates with the MT (states 1 and 3, respectively). ATP binds to the KIF3A (state 2) or KIF3C (state 4). The steps after ATP binding, which are collapsed in this model, consist of the motor undergoing a series of transitions that include neck-linker docking, forward movement, and MT association of the trailing head, followed by ADP release. Subsequently, ATP hydrolysis occurs followed by phosphate release with dissociation of the new trailing head (transitions 2-3 for the KIF3A pathway and transitions 4-1 for the KIF3C pathway). The kinesin can then bind another ATP molecule to undergo another turnover of ATP, or alternatively, phosphate release can result in detachment of the dimer from the MT (state 5). The rate constants associated with these steps in the cycle are defined in Table 2. MT, microtubule.

Recent results from Bensel *et al.* (22) evaluated the stepping kinetics of KIF3AC in the absence of load by interferometric scattering and under load by optical trapping. Their results indicate that the stepping durations of KIF3AC single molecules are best described by a single rate without apparent asymmetry. To evaluate these results, we pursued experiments to measure the presteady-state kinetics of phosphate release because the transitions associated with phosphate release coupled with motor head dissociation from the MT represent the duration of each step of KIF3A and KIF3C in the processive run. These experiments were followed by computational modeling of the KIF3AC fluorescence transients. The results show that for KIF3AC, KIF3A and KIF3C each retain their intrinsic ATP binding and hydrolysis kinetics. However, KIF3A activates the KIF3C kinetics of phosphate release coupled to dissociation from MT. The modeling also shows that KIF3A activates the rate of KIF3C MT association followed by ADP release (E2A–E4A). In addition, the results indicate that phosphate release coupled with motor dissociation from the MT is rate limiting for the ATPase cycle and predicts that within a processive run, KIF3A and KIF3C will exhibit similar step kinetics.

## Results

Figure 1*A* shows a detailed KIF3AC stepping cycle that can begin by either the KIF3A or the KIF3C motor domain. KIF3AC in solution is designated the E0 state. To begin the processive run, KIF3AC collides with the MT by either the KIF3A head (E0A) or the KIF3C head (E0C) followed by ADP release to form the E1A or E1C intermediate. ATP binding is followed by a series of structural transitions, which include neck linker docking and movement of the trailing head forward to the next MT-binding site (E2–E3). ADP is released from the leading head, and the neck linkers become strained with both heads tightly bound to the MT (E3–E4). ATP is hydrolyzed at the trailing head followed by phosphate release coupled to detachment of the trailing head from the MT (E5–E1) to form the ATP-waiting state (E1C or E1A). If the KIF3A head were to collide with the MT first (E1A–E5A), then the subsequent ATP-binding event occurs at E1C, its partner head. Similarly, if KIF3C collides with the MT initially (E1C–E5C), the second ATP binds at E1A. Here, we present the kinetics of phosphate release after ATP hydrolysis for KIF3AC, KIF3AA, and KIF3CC and use computational modeling to probe the steps of phosphate release coupled to motor head dissociation from the MT (E5A–E1C or E5C–E1A).

### Phosphate-release kinetics

**Figure 2:**
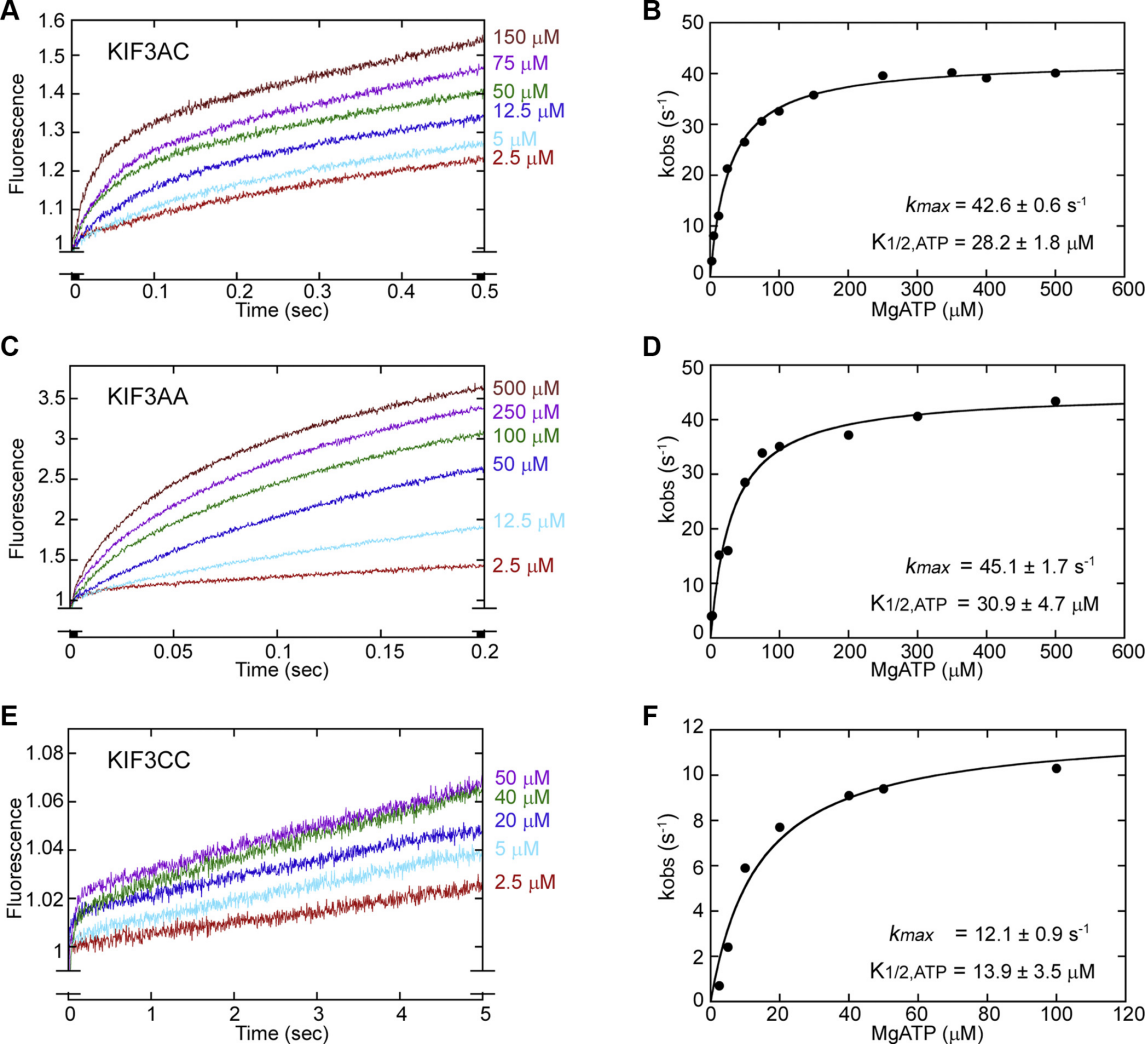
**KIF3AC, KIF3AA, and KIF3CC phosphate-release kinetics.** The preformed MT⋅KIF3 complex (2 μM MT plus 0.5 μM KIF3 dimer), 5 μM MDCC–PBP, 150 μM MEG, and 0.05 U/ml PNPase in the first syringe were rapidly mixed with varying concentrations of MgATP in the second syringe plus either 250 mM KCl (KIF3AC) or 300 mM KCl (KIF3AA and KIF3CC), 150 μM MEG, and 0.05 U/ml PNPase. The fluorescence transients that represent phosphate release by KIF3AC (*A*), KIF3AA (*C*), and KIF3CC (*E*) are presented. A double exponential function was fit to each KIF3AC transient to determine the initial exponential rates of phosphate release. Alternatively, because of the challenge of resolving two phases in the KIF3AA and KIF3CC fluorescence transients, a single exponential function plus a linear term was used to provide the initial exponential rate of phosphate release for the homodimers. These initial exponential rates were plotted as a function of MgATP concentration for KIF3AC (*B*), KIF3AA (*D*), and KIF3CC (*F*). A hyperbolic fit to the data provided the maximum rate constant of phosphate release and the *K*_1/2,ATP_. MDCC, *N*-[2-(1-maleimidyl)ethyl]-7-(diethylamino) coumarin-3-carboxamide; MEG, 7-methylguanosine; MgATP, magnesium ATP; MT, microtubule; PBP, phosphate-binding protein; PNPase, purine nucleoside phosphorylase.

The presteady-state kinetics of phosphate release was measured directly using the *N*-[2-(1-maleimidyl)ethyl]-7-(diethylamino) coumarin-3-carboxamide (MDCC)–phosphate-binding protein (PBP) fluorescence assay developed by Webb *et al.* ((23); Experimental procedures). The MT⋅KIF3AC complex (Fig. 1*A*; E1A or E1C) was preformed and rapidly mixed with magnesium ATP (MgATP) plus potassium chloride. ATP binds to the active site of the motor domain, is hydrolyzed, and phosphate is subsequently released. When phosphate is released from the active site of the motor domain to the solution, it is bound rapidly and tightly by MDCC–PBP resulting in a fluorescence enhancement as observed in the transients in Fig. 2. Note that the potassium chloride salt does not affect the first ATP turnover but weakens the rebinding of the KIF3⋅ADP head to its next MT-binding site for the second ATP turnover (Fig. 1*A*; E1C–E3C or E1A–E3A). Therefore, the added salt can improve the separation of the first ATP turnover from subsequent ATP turnovers in the transients (24, 25).

For KIF3AC (Fig. 2*A*), the transients reveal a fluorescence enhancement as a function of time that is biphasic with an initial fast exponential phase corresponding to the first ATP turnover followed by a slower phase that we attribute to subsequent turnovers. There is both an increase in fluorescence amplitude and the observed rate as a function of ATP concentration. A double exponential function was fit to each transient, and the observed rates of the initial phase were plotted as a function of ATP concentration (Fig. 2*B*). The hyperbolic fit of these data revealed a maximum observed rate of phosphate release at 42.6 s^−1^ with *K*_1/2, ATP_ = 28.2 μM. Figure 2, *C* and *D* shows that the phosphate-release kinetics for KIF3AA was also biphasic, yet the first and second phases were not well separated even at the shorter time domain for data collection at 0.2 s. Therefore, a single exponential function plus a linear term was used to fit the KIF3AA transients, and the exponential rate of the initial fast phase was plotted as a function of ATP concentration (Fig. 2*D*). The hyperbolic fit to the data revealed a maximum rate of 45.1 s^−1^ with *K*_1/2, ATP_ = 30.9 μM. In contrast, when the experiment was repeated for KIF3CC, the results were quite different. The observed rate of phosphate release was slower with *k*_max_ = 12.1 s^−1^ and *K*_1/2, ATP_ = 13.9 μM. However, the 12.1 s^−1^ rate constant for phosphate release for KIF3CC was much faster than predicted by the steady-state ATPase *k*_*cat*_ and the single-molecule velocity, both at ∼1 s^−1^ (Table 1).

Interestingly, the rate of phosphate release and the *K*_1/2, ATP_ for KIF3AC (Fig. 2*B*) were more similar to KIF3AA (Fig. 2*D*) than KIF3CC (Fig. 2*F*). Because half of the KIF3AC dimer is bound to the MT by KIF3A and half by KIF3C during the first ATP turnover (21), the phosphate release rates of both heads contribute to the measured KIF3AC phosphate release rate of 42.6 s^−1^. If KIF3A and KIF3C were to retain their intrinsic phosphate release rates of 45.1 and 12.1 s^−1^, respectively, the measured KIF3AC phosphate release rate would be expected to be slower than 42.6 s^−1^. However, because phosphate release occurs via two alternate pathways for each ATP turnover, the KIF3AC phosphate release rate would not necessarily resemble that of KIF3CC at 12 s^−1^. In order for KIF3AC to achieve a phosphate release rate of 42.6 s^−1^, KIF3C must be accelerated by KIF3A within KIF3AC. Moreover, the rates of the second phase for KIF3AC as well as KIF3AA and KIF3CC did not show an ATP concentration dependence. These rates were quite slow at 2 to 4 s^1^, which we attribute to the multiple ATP turnovers of the second phase and the added salt used that affects subsequent turnovers (24, 25, 26, 27). The experimentally determined phosphate release results for KIF3AA, KIF3CC, and KIF3AC helped guide the KIF3AC model derivation and simulation.

### Model assumptions

Before the development of the model, several assumptions were made. First, previous modeling work showed that there is approximately a 50% probability that KIF3A or KIF3C can begin the processive run (21). Therefore, the transitions that occur when KIF3A and KIF3C begin the processive run must be modeled in parallel. Second, the binding of ATP results in an ATP-promoted isomerization that is 81 s^−1^ followed by ATP hydrolysis at 69 s^−1^ (Table 1). Both these steps are fast; therefore, the nucleotide-promoted transitions after ATP binding that culminate with phosphate release and dissociation from the MT can be collapsed. Third, because MDCC–PBP can bind rapidly and tightly to phosphate, the release of phosphate is assumed to be coupled to its binding to MDCC–PBP, which results in an enhancement of fluorescence. Therefore, the relative change in fluorescence can be correlated with the time-dependent change in the concentration of phosphate released from the active site (Fig. 1*A*; E5A–E1C, E5C–E1A).

It is important to note that ATP can bind to a single head to begin the processive run, whereas the partner head participates in some of the subsequent transitions of the stepping cycle. For example, once the processive run begins with ATP binding, the partner head moves forward 16 nm, associates with the MT, and releases ADP (Fig. 1*A*; E2–E4). Hereafter, when describing the model, the two parallel pathways will be referred to as the KIF3A and KIF3C pathways, where each pathway begins with ATP binding by KIF3A and KIF3C, respectively.

### The computational model

**Figure 3:**
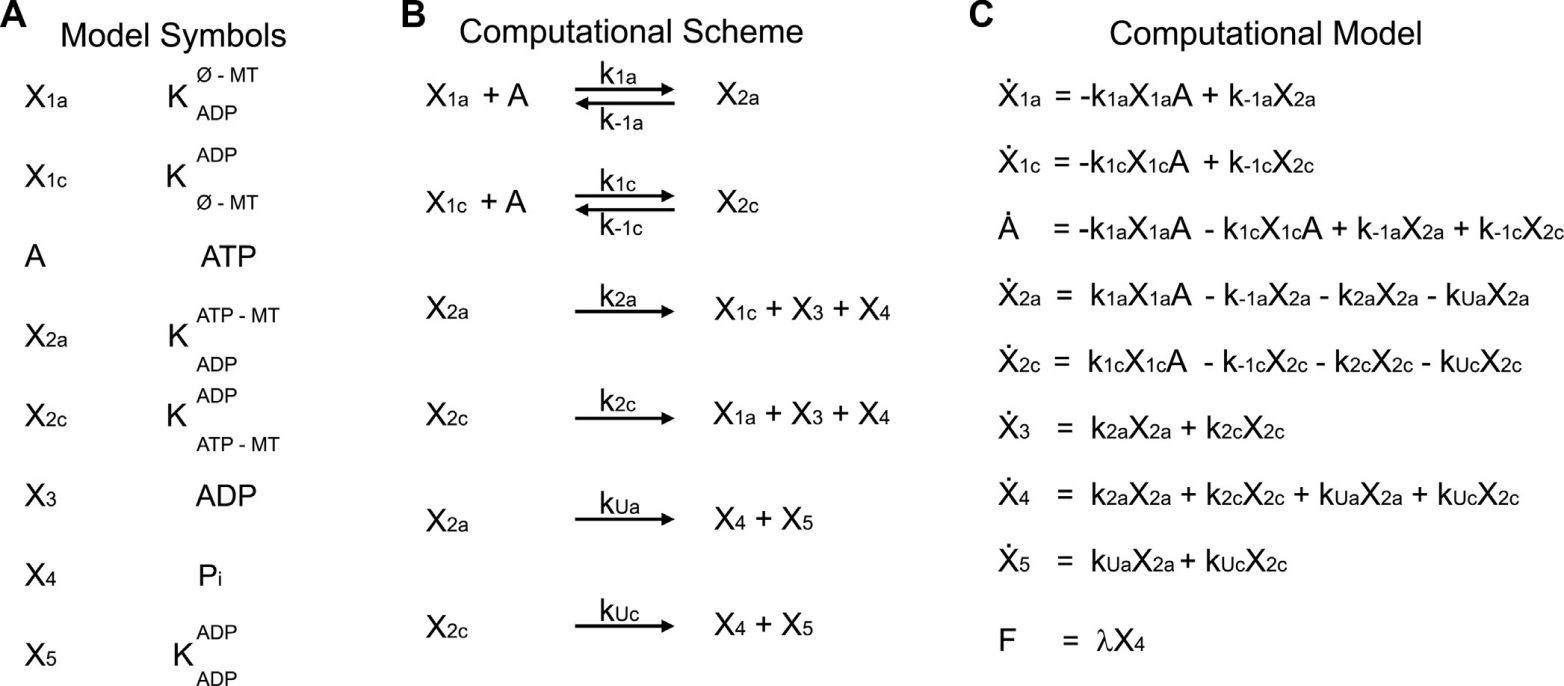
**Model symbols, computational scheme, and computational model.***A,* the model symbols are indicated. K represents KIF3, and the superscript and subscript species represent nucleotide- and MT-binding states for each head. The symbol ø represents no nucleotide bound, and MT represents a MT-bound motor head. *B*, the computational scheme consists of six reactions as illustrated in the legend to Fig. 1*B*. First, ATP binds to the KIF3A or KIF3C heads (reactions 1 and 2, respectively). After ATP binding, there is a combined series of transitions where neck-linker docking occurs followed by the forward movement of the tethered trailing head to the next MT-binding site and ADP release. ATP bound to the rear head is subsequently hydrolyzed followed by coupled phosphate release and dissociation (reactions 3 and 4 for the KIF3A and KIF3C pathways, respectively). Alternatively, after ATP binding, the KIF3AC heterodimer can enter into a state that detaches completely from the MT (reactions 5 and 6 for the KIF3A and KIF3C pathways, respectively). *C,* the computational model consists of eight ordinary differential equations, which represent the change in concentration of the different KIF3AC intermediates and substrates. MT, microtubule.

To better understand how ATP association and the nucleotide-promoted transitions thereafter regulate the velocity of KIF3AC, we developed and implemented a nonlinear ordinary differential equation (ODE) model to simulate the phosphate-release kinetics captured by the fluorescence transients presented in Fig. 2*A*. Because there is an equal probability of KIF3A and KIF3C beginning the processive run of KIF3AC, the model begins with ATP binding to KIF3A for the KIF3A pathway (Fig. 1*B*; transition 1-2 clockwise) or KIF3C for the KIF3C pathway (Fig. 1*B*; transition 3-4 clockwise). For these reactions, *k*_1a_ and *k*_1c_ represent the ATP-binding rate constants, whereas *k*_−1a_ and *k*_−1c_ represent the ATP off rates for KIF3A and KIF3C, respectively. After ATP binding to the leading head, the trailing head moves forward to the next MT-binding site, collides with the MT, and releases ADP (transitions 2-3 clockwise for KIF3A and transitions 4-1 clockwise for KIF3C). ATP is subsequently hydrolyzed, phosphate is released, and the trailing head dissociates from the MT, where KIF3AC returns to an ATP-waiting state (Fig. 1*B*; transitions 2-3 and 4-1 clockwise). Because the ATP-promoted isomerization and ATP hydrolysis have been shown to be fast steps for KIF3AC, these transitions can be collapsed with phosphate release and trailing head dissociation. The rate constants for these combined transitions are represented as *k*_2a_ for the KIF3A pathway and *k*_2c_ for the KIF3C pathway. In order for the kinesin to enter into an ATP-waiting state, at least one head must be tightly bound to the MT. If KIF3AC were to enter into a state where both heads are weakly bound to the MT (*i.e.*, where both heads are in an ADP bound state), the dimer would dissociate from the MT, and the processive run would end. This would require ATP hydrolysis and phosphate release to occur before the trailing head has bound the next MT site. In the model, the rates representing KIF3AC dimer dissociation after ATP hydrolysis and phosphate release by KIF3A and KIF3C, respectively, are represented by *k*_Ua_ and *k*_Uc_ in Fig. 1*B* (transitions 2-5; right to left for KIF3A and transitions 4-5; left to right for KIF3C). Finally, the fluorescence scales linearly with the concentration of released phosphate; therefore, the concentration of released phosphate can be converted to fluorescence for the simulations using the scaling constant λ (Fig. 3).

**Figure 4:**
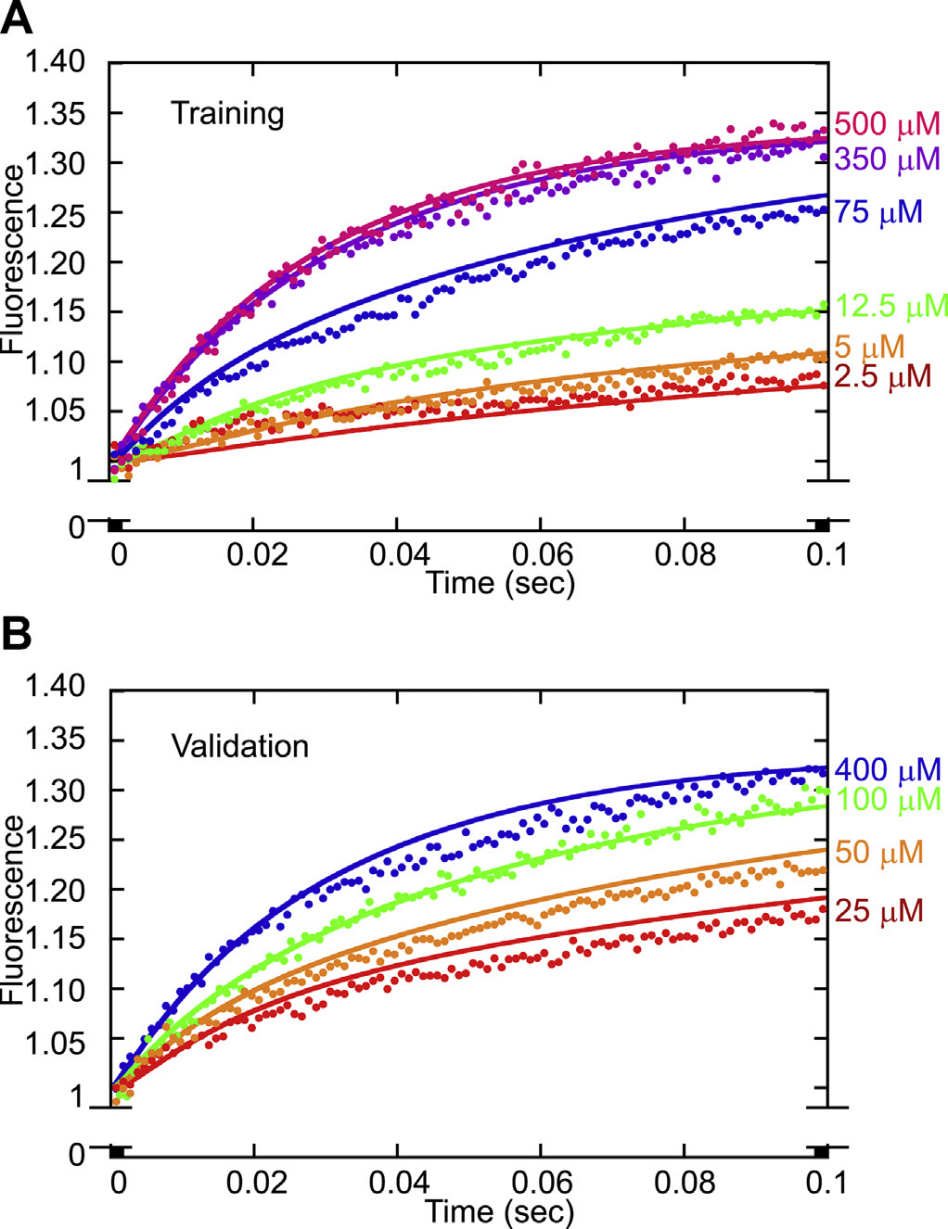
**KIF3AC simulations of the phosphate release transients.** The phosphate release fluorescence transients were simulated using the computational scheme presented in the legend to Fig. 1*B*. The solid lines correspond to the simulations, and only 100 points of the fluorescence transients are presented for clarity. Because all subsequent turnovers after the first turnover cannot be resolved from the model, all fluorescence transients were simulated up to 100 ms to represent the first turnover for each head of the heterodimer. *A,* representative fluorescence transients used for training and corresponding simulations. *B,* fluorescence transients used for validation and corresponding simulations.

Once the outline of the model was conceptualized, the model equations were derived. The eight ODEs presented in Fig. 3*C* represent the change in the concentration of the nucleotide and kinesin states as a function of time. The model also contained nine parameters. Previously published single-molecule and presteady-state kinetics experiments guided the initial assignment of values for these parameters. However, the values for several of them were re-estimated. In addition, the equation for fluorescence (F) represents the fluorescence output. Finally, the modeling procedure required the fluorescence data for different concentrations of ATP to be allocated for training or validation. The model was fit to the data set allocated for training to determine the estimated parameters representing rate constants for various transitions within the KIF3AC stepping cycle. Fluorescence data assigned for validation were used to assess the model performance (*i.e.*, the ability of the model to fit the data not used for training). The simulations presented in Figure 4 contain the data allocated for training (Fig. 4*A*) and validation (Fig. 4*B*), and the solid lines correspond to the simulations. Additional details on the computational methods can be found in Experimental procedures.

### Model assumptions and experimentally determined rate constants guided kinetic parameter set selection for estimation

To prevent overfitting of the model, it was necessary to minimize the number of estimated model parameters. Parameters were first identified that could be fixed to values of previously determined kinetic rate constants for KIF3AC, and model parameter values with the most uncertainty were selected for estimation. Because ATP-binding rate constants were previously determined for KIF3AA, KIF3CC, and KIF3AC, the ATP-binding kinetic parameters were good candidates for being set to fixed values for the modeling. Likewise, this work provided phosphate release rates for KIF3AA, KIF3CC, and KIF3AC. Therefore, the experimentally determined phosphate release rates were assigned to the KIF3AC model parameters representing the combined steps resulting in phosphate release and the regeneration of the ATP-waiting state. A preliminary hypothesis was that heterodimerization would likely impact the combined steps resulting in dimer dissociation (Fig. 1*B*; *k*_Ua_ and *k*_Uc_); therefore, these model parameters were selected for estimation.

### KIF3A and KIF3C retain their intrinsic catalytic properties for ATP association

Before settling on fixed values for the model parameters representing ATP binding, preliminary simulations were run to test whether KIF3AC achieves an ATP-binding rate of 11 μM^−1^s^−1^ by KIF3A and KIF3C binding to ATP with equivalent rates. Alternatively, the rate of ATP binding by KIF3A could exceed that of KIF3C. To test these two possibilities, preliminary simulations were run with the ATP-binding rates for KIF3A and KIF3C fixed at 11 μM^−1^s^−1^. These simulations resulted in poor model fits to the fluorescence transients. Furthermore, simulations were run by fixing KIF3A to the KIF3AA ATP-binding rate 16 μM^−1^s^−1^ or to the KIF3AC ATP-binding rate of 11 μM^−1^s^−1^ (Table 1) while estimating the corresponding model parameter for KIF3C. In either case, the KIF3C ATP-binding model parameter resembled that of KIF3CC at 0.68 μM^−1^s^−1^ (Table 1). Therefore, going forward, the KIF3A and KIF3C ATP-binding parameters were fixed at 11 and 0.68 μM^−1^s^−1^, respectively. This suggests that both heads retain their relative intrinsic ATP association kinetics. Because ATP association by KIF3AC was previously determined to be fast at ∼11 μM^−1^s^−1^, ATP binding is likely dominated by the intrinsic catalytic properties of KIF3A and must occur with a rate of at least 11 μM^−1^s^−1^. In addition, the model predictions for the ATP off rates showed that the simulations best fit the data when the off rates were fast at 126 and 63.7 s^−1^ for both KIF3A and KIF3C, respectively (Table 2).

**Table 2:** KIF3AC model parameter estimation

Parameter	Units	Set/estimated	Value	Lower limit	Upper limit
*k*_1a_	μM^−1^s^−1^	Set	11		
*k*_−1a_	s^−1^	Estimated	126	110.4	133.8
*k*_1c_	μM^−1^s^−1^	Set	0.68		
*k*_−1c_	s^−1^	Estimated	63.7	60.1	69.9
*k*_2a_	s^−1^	Set	45.1		
*k*_2c_	s^−1^	Set	12.1		
*k*_Ua_	s^−1^	Estimated	44.5	39.9	46.7
*k*_Uc_	s^−1^	Estimated	36.3	34.5	40.4
λ	—	Estimated	0.86	0.8519	0.8648
Training MSE	2.01 × 10^−4^		Validation MSE	2.06 × 10^−4^	

MSE, mean-squared error.

### The processive KIF3AC model suggests that KIF3A accelerates KIF3C-coupled phosphate release and dissociation

In the computational model, ATP hydrolysis and phosphate release can either result in KIF3AC entering into a single-head bound ATP-waiting state (Fig. 1*B*; transition 2-3 for the KIF3A pathway or 4-1 for the KIF3C pathway; clockwise) or detaching from the MT (Fig. 1*B*; transition 2–5 for the KIF3A pathway; right to left or 4–5 for the KIF3C pathway; left to right). Therefore, the measured phosphate-release kinetics for KIF3A or KIF3C must be attributed to a combination of phosphate release from these two parallel pathways: phosphate release before dimer dissociation from the MT and phosphate release to generate the one-head bound ATP-waiting state. The model predicted (Table 2) that for the KIF3A pathway, the rate for combined steps including ATP hydrolysis and phosphate release that resulted in dimer detachment *k*_Ua_ was 44.5 s^−1^ (95% confidence interval [95% CI], 39.9, 46.7 s^−1^), whereas the rate for ATP hydrolysis and phosphate release resulting in the generation of the single-head bound ATP-waiting state *k*_2a_ was 45.1 s^−1^. The equal phosphate release rates for these two parallel paths suggest that after ATP hydrolysis (Fig. 1*B*; intermediate 2), the intermediate has an equal probability of either detaching from the MT or regenerating the single-head bound ATP-waiting state after phosphate release. In addition, within KIF3AC, the model predicts that KIF3A should achieve a phosphate release rate of between 44 and 45 s^−1^ (Table 2).

Unlike KIF3A, the model predicted that after ATP binding to KIF3C (Table 2), the combined steps resulting in phosphate release and dimer detachment (*k*_Uc_) occur at a rate of 36.3 s^−1^ (95% CI, 34.5, 40.4 s^−1^). The model also predicted that the combined steps that result in regeneration of the ATP-waiting state occur at a rate of 12.1 s^−1^ (*k*_2c_). Although these rates predicted by the modeling are ∼3-fold different, it is important to note that these rates are dependent on the probability of the MT-bound KIF3C intermediate (Fig. 1*B*; intermediate 4) proceeding down these two parallel pathways. Therefore, a rate of 12.1 s^−1^ for *k*_2c_ and 36.3 s^−1^ (95% CI, 34.5, 40.4 s^−1^]) for *k*_Uc_ may represent apparent rates dependent on probability, but because the same transitions are collapsed, the actual rates for the two paths cannot be easily determined through the modeling.

However, these results do suggest that within the KIF3AC processive run, after ATP binding, ATP hydrolysis and phosphate release by KIF3C results in a greater population of dimer detachment from the MT. Although determining a rate of phosphate release by KIF3C from these results is nontrivial, a rate of 36.3 s^−1^ or faster may better represent the combined steps resulting in phosphate release rate of KIF3C. Considering the rate of KIF3AC phosphate release was determined to be 42.6 s^−1^ experimentally and KIF3A phosphate release determined through the modeling was ∼45 s^−1^, KIF3C would be expected to undergo the combined transitions resulting in released phosphate at a rate of ∼40 s^−1^. Importantly, the computational modeling complements the experimental work by demonstrating that KIF3A accelerates KIF3C phosphate release.

### The velocity of processive stepping is not controlled by the intrinsic catalytic properties of KIF3C

The results from this work also show that the stepping of KIF3AC is determined by heterodimerization of the KIF3A and KIF3C polypeptides. Previously, the single-molecule velocity was reported to be 186 nm/s (28). This velocity is significantly faster than that of KIF3CC at 7.5 nm/s and suggests that the intrinsic catalytic properties of KIF3C cannot limit the velocity of KIF3AC (4, 15). These results can be confirmed by calculating the velocity from the experimental and modeling results. In the computational model presented in this work, a step duration is either the time at which the kinesin goes from one single-head bound ATP-waiting state to the next (*i.e.,* where the two heads become interchanged) or from the single-head bound ATP-waiting state to dimer dissociation within a given ATP turnover. The transitions that comprise a step by KIF3A or KIF3C are ATP binding and the combined transitions resulting in phosphate release, regardless of whether kinesin detaches from the MT or regenerates the single-head bound waiting state. Therefore, to determine the dwell times for KIF3A and KIF3C, the duration of ATP binding and the combined steps resulting in phosphate release must be summed for the two heads. The model predicted that KIF3A and KIF3C bind to ATP with a rate constant of 11 μM^−1^s^−1^ and 0.68 μM^−1^s^−1^, respectively. Assuming a high concentration of ATP (>500 μM) with reversible binding, the rates of ATP binding are fast at 5626 s^−1^ and 404 s^−1^ for KIF3A and KIF3C, respectively. From the modeling and experimental work, the rates for the combined transitions resulting in phosphate release were ∼45 s^−1^ for KIF3A and ∼40 s^−1^ for KIF3C. The calculated transit time for KIF3A would be 1/5626 s^−1^ + 1/45 s^−1^ or 0.0224 s and for KIF3C would be 1/404 s^−1^ + 1/40 s^−1^ or 0.0275 s. Therefore, the total duration for a KIF3A and KIF3C step would be 0.0224 s + 0.0275 s or 0.050 s. Considering that each step by KIF3AC results in an 8-nm displacement along the MT, the velocity of KIF3AC would be 320 nm/s (16 nm/0.050 s). Together, the modeling and experimental results confirm that KIF3C would not limit the rate of stepping of KIF3AC. Moreover, KIF3A accelerates KIF3C such that both heads achieve similar rates of stepping.

### Goodness of fit of the model to the experimental data

The goodness of fit of the model to the experimental data was evaluated by computing a mean-squared error (MSE) value. Deviations of the simulation fits to the experimental data were attributed to the high sensitivity of the experiment and the fact that the fluorescence transients were from a wide range of MgATP concentrations, 2.5 to 500 μM MgATP. For example, the model slightly overshoots the fluorescence transients for some MgATP concentrations, yet undershoots others. Despite these minor discrepancies in the model fit to the data, the training and validation MSE of 2 &times 10^−4^ are consistent in magnitude with our previous modeling work, which suggests that the fits to the data are reasonable (21).

## Discussion

The computational modeling of the phosphate-release kinetics presented in this work served to provide additional insight into how heterodimerization regulates the motility properties of KIF3AC. In the ATP-binding step, the model predicted that KIF3A and KIF3C retained their intrinsic ATP-binding kinetics within KIF3AC. Because the ATP-binding rates of KIF3A and KIF3C are ∼20-fold different, the model would also predict that both heads would likely undergo asymmetric stepping under low ATP conditions. In addition, for the combined transitions consisting of ATP-promoted isomerization, ATP hydrolysis, and phosphate release, the model differentiated between KIF3AC resulting in a single head-bound ATP-waiting state (Fig. 1*B*; transitions 2-3 and 4-1; clockwise) and KIF3AC detached from the MT (Fig. 1*B*; transitions 2-5; right to left and 4-5; left to right). Both populations of kinesins would contribute to single observed rates of ∼45 s^−1^ and ∼40 s^−1^, corresponding to the rates of the combined transitions resulting in phosphate release for the KIF3A and KIF3C pathways, respectively. The transit times for each step, which is the sum of the duration of ATP binding and the combined transitions resulting in phosphate release, were 0.0224 s for KIF3A and 0.0275 s for KIF3C. These transit times suggest that KIF3A steps at a rate of 44.6 s^−1^ (1/[0.0224 s]), whereas KIF3C steps at a rate of 36.4 s^−1^ (1/[0.0275 s]), demonstrating that KIF3AC takes alternating steps with approximately symmetrical stepping kinetics.

The results also suggest that both heads do not retain their relative intrinsic properties for each step within the combined transitions from ATP-promoted isomerization through phosphate release and dissociation. For example, the model predicts that ADP release by KIF3C within the processive run must greatly exceed ADP release during entry into the processive run at 7.5 s^−1^ (Table 1). These findings also indicate that heterodimerization does alter the kinetics of KIF3A and KIF3C from their intrinsic properties.

These results are also in agreement with the work by Bensel *et al.* (22), where they found that fitting KIF3AC dwell time cumulative distribution data generated from interferometric scattering microscopy experiments could only be statistically justified with a single-exponential function rather than the sum of two exponentials. Moreover, their step trajectory analysis showed that alternating steps had similar step lifetimes. Significantly though, the optical trap results revealed a mechanism by which KIF3AC achieves its motility properties. KIF3AC has a single observed stepping rate in the presence and absence of load and detaches rapidly under load. Moreover, both assisting load and KIF3A accelerate the stepping kinetics of KIF3C, yet KIF3CC does not stall but detaches from the MT under hindering load. MT gliding assays revealed that an equal mixture of KIF3AA and KIF3CC achieve the same velocity of MT gliding as KIF3AC. The authors conclude that the stepping of KIF3C can be activated by KIF3A in a strain-dependent manner and that the mechanochemical properties of KIF3AC are due to the strain-dependent kinetics of KIF3A and KIF3C (22).

The results presented here and those from Bensel *et al.* (22) indicate that KIF3AC is an unconventional processive kinesin, yet the question becomes how do these mechanochemical properties enable the physiological function of KIF3AC in neurons? Because neurons are highly polarized cells that depend on selective transport of specific vesicles to axons and dendrites (1, 29, 30, 31), the first thought is that KIF3AC transports specific cargoes yet to be identified. In this context, KIF3AC is well suited to function as a transporter in larger teams of motors rather than a few (32) or for cooperative transport with other faster motors as observed for KIF3AB–KAP and KIF17 (17, 33, 34, 35). Note though that KIF3AC labels vesicles that are restricted to the somatodendritic region of hippocampal neurons, and these seldom undergo long-range transport (6). These observations suggest another role. The motile properties of KIF3AC are also well suited for a tethering role in dendrites where MTs exhibit an anti-parallel orientation (36, 37), which would allow KIF3AC motors on the vesicle surface to bind MTs of opposite orientation and remain stationary unless activated. Alternatively, KIF3AC may have more traditional transport roles during early development and prior to specification of the axon (38). These are exciting questions for future experimentation.

In conclusion, the mathematical modeling predicted that heterodimerization can regulate the motility properties of KIF3AC. The results indicate that the rate-limiting step in the ATPase pathway is not controlled specifically by neither the intrinsic kinetics of KIF3C nor the ADP release by KIF3C. Rather the results indicate that within the processive run, it is the coupled steps of phosphate release and motor head dissociation that determine the velocity of stepping. Finally, the modeling work confirmed that during the processive run, KIF3A and KIF3C may prove to be not so different after all.

## Experimental procedures

### Kinesin-2 KIF3 plasmid design and expression

The *Mus musculus KIF3A* and *KIF3C* plasmids for KIF3AC, KIF3AA, and KIF3CC protein expression were described in detail previously (15). For KIF3AC, KIF3A contained a native N-terminal motor domain, neck-linker, and helix α7 (Met^1^-Leu^374^), followed by an end binding protein 1 dimerization domain (DFYFGKLRNIELICQENEGENDPVLQRIVDILYATDE), and a tobacco etch virus (TEV) protease-cleavable StrepII tag (TTSENLYFQGASNWSHPQFEK). Predicted molecular weight (MW) is 48,559. KIF3C contained a native N-terminal motor domain, neck-linker, and helix α7 (Met^1^-Leu^396^), followed by an end binding protein 1 dimerization domain (DFYFGKLRNIELICQENEGENDPVLQRIVDILYATDE), and a TEV protease-cleavable His_8_ tag (TTSENLYFQGASHHHHHHHH). Predicted MW is 49,759. KIF3A of homodimeric KIF3AA was similarly designed with the N-terminal KIF3A motor domain (Met^1^–Leu^374^), followed by an end binding protein 1 dimerization domain (DFYFGKLRNIELICQENEGENDPVLQRIVDILYATDE), and a TEV protease cleavable His_8_ tag (TTSENLYFQGASHHHHHHHH). Predicted MW is 48,502.

### KIF3 protein expression

KIF3AC, KIF3AA, and KIF3CC were expressed in *Escherichia coli* cell line BL21-CodonPlus (DE3)**-**RIL cells as described previously (15, 18, 20, 21). The KIF3AC heterodimers resulted from cotransformation of the *KIF3A* and *KIF3C* plasmids, each with different antibiotic resistance (ampicillin, *KIF3A*; kanamycin, *KIF3C*). Transformed cells were selected on LB agar plates containing 100 μg/ml ampicillin, 50 μg/ml kanamycin, and 10 μg/ml chloramphenicol. Colonies were selected from the triple antibiotic plates and subsequently inoculated in liquid LB containing 100 μg/ml ampicillin, 30 μg/ml kanamycin, and 10 μg/ml chloramphenicol. Cultures were grown at 37 °C at 225 rpm until they reached *A*_600_ of ∼0.4. The cultures were chilled in an ice bath to 16 °C, and protein expression was induced by the addition of 0.1 mM isopropyl *β*-*D*-1-thiogalactopyranoside. Protein expression continued at 16 °C with shaking at 185 rpm for ∼15 h. The cells were harvested by centrifugation and resuspended in lysis buffer at 10 ml/g cells. Lysis buffer is 10 mM sodium phosphate buffer, pH 7.2, 300 mM sodium chloride (NaCl), 2 mM MgCl_2_, 0.1 mM EGTA, 10 mM PMSF, 1 mM DTT, 0.02 mM ATP, and 30 mM imidazole. KIF3AA and KIF3CC were expressed with the appropriate antibiotic selection following the same protocol.

### Purification of KIF3 dimers

The purification strategy of these kinesins has been described in detail previously (15, 18, 20, 21). Briefly, an aliquot of the cell suspension, ∼10 g/100 ml lysis buffer supplemented with 0.1 mg/ml lysozyme, was incubated with gentle stirring in an ice bath for 45 min. The cells were lysed by three replicates of freezing in liquid nitrogen and thawing in a 37 °C water bath. After clarification by centrifugation, the supernatant was applied to a pre-equilibrated HisTrap fast flow Ni^2+^-nitrilotriacetic acid column (GE Healthcare). Column-binding buffer: 20 mM sodium phosphate, pH 7.2, 300 mM NaCl, 2 mM MgCl_2_, 0.1 mM EGTA, 0.02 mM ATP, and 30 mM imidazole. After loading, the column was washed with column buffer to return the absorbance to baseline. The His_8_-tagged kinesins were eluted with a linear gradient of 30 to 300 mM imidazole in the Ni^2+^-nitrilotriacetic acid column-binding buffer, pH 7.2. The fractions enriched in the specific His8-tagged KIF3 were identified through SDS-PAGE. For KIF3AA and KIF3CC, these fractions were pooled, concentrated, and dialyzed in 20 mM 4-(2-hydroxyethl)-1-piperazineethanesulfonic acid, pH 7.2 with potassium hydroxide (KOH), 0.1 mM ED TA, 0.1 mM EGTA, 5 mM magnesium acetate, 50 mM potassium acetate, 1 mM DTT plus NaCl in sequential buffers containing 300, 200, and 100 mM NaCl with the final dialysis buffer plus 5% sucrose (w/v).

For the KIF3AC heterodimer, the fractions from the HisTrap fast flow Ni^2+^- nitrilotriacetic acid column enriched in both KIF3A with the StrepII tag and KIF3C with the His_8_ tag were transferred to a pre-equilibrated StrepTrap HP column to select for the C-terminal Strep-tagged KIF3A. The StrepTrap HP column-binding buffer is 20 mM sodium phosphate, pH 7.2, 300 mM NaCl, 2 mM MgCl_2_, 0.1 mM EGTA, 1 mM DTT, and 0.02 mM ATP. Once loaded, the StrepTrap column was washed with excess column buffer to return the protein absorbance to baseline, followed by elution with the StrepII column buffer plus 2.5 mM desthiobiotin.

Fractions were analyzed by SDS-PAGE to identify fractions that contained a 1:1 ratio of KIFA to KIF3C polypeptides. These fractions were subsequently pooled, concentrated, and dialyzed at 4 °C in 20 mM sodium phosphate buffer, pH 7.2, 2 mM MgCl_2_, 0.1 mM EGTA, 1 mM DTT, plus NaCl in sequential dialysis steps: 200 and 100 mM NaCl. The final dialysis step was a buffer at 20 mM 4-(2-hydroxyethl)-1-piperazineethanesulfonic acid, pH 7.2 with KOH, 100 mM NaCl, 0.1 mM EGTA, 0.1 mM EDTA, 5 mM magnesium acetate, 50 mM potassium acetate, and 1 mM DTT, plus 5% sucrose (w/v).

The proteins were concentrated, clarified by ultracentifugation, and aliquoted before freezing in liquid nitrogen and stored at −80 °C. For the experiments presented here, the purification tags were not cleaved. The predicted MWs of the dimeric KIF3 proteins based on amino acid residue sequence are as follows: KIF3AC at 98,318, KIF3AA at 97,004, and KIF3CC at 99,518.

### Purification of MDCC–PBP

PBP was purified and labeled with coumarin MDCC as described (23) with modifications (39).

### Experimental conditions

All experiments presented in this article were performed at 25 °C using ATPase buffer (20 mM 4-(2-hydroxyethl)-1-piperazineethanesulfonic acid, pH 7.2 with KOH, 5 mM magnesium acetate, 0.1 mM EDTA, 0.1 mM EGTA, 50 mM potassium acetate, 1 mM DTT, plus 5% sucrose). Preparation of the MTs for each experiment required the bovine brain tubulin to be cold depolymerized, clarified, and then polymerized using 1 mM GTP at 37 °C. The polymerized MTs were subsequently stabilized using 40 μM paclitaxel. The concentration of MTs was determined using a Lowry assay with bovine serum albumin as the standard. MT concentrations reported represent the concentration of paclitaxel-stabilized *α*/*β* tubulin.

### Phosphate-release kinetics

The kinetics of phosphate release was determined using a coupled-assay system with MDCC–PBP as described (23, 24, 25, 40, 41). The MDCC fluorophore was excited at 436 nm with fluorescence emission detected at 474 nm by using a 450-nm cutoff filter. Contaminating free phosphate was removed from the solutions, and the stopped-flow observation cell using a Pi Mop, consisting of 0.05 units/ml purine nucleoside phosphorylase (PNPase) and 150 μM 7-methylguanosine (7-MEG) in ATPase buffer. Note that the concentration of PNPase was adjusted to achieve a rate of phosphate removal at ∼0.005 s^−1^ to avoid competition of the Pi Mop with MDCC–PBP.

For the stopped flow experiments, the preformed MT⋅KIF3 complex (2 μM KIF3 dimer), 5 μM MDCC–PBP, 150 μM MEG, and 0.05 units/ml PNPase were rapidly mixed with varying concentrations of MgATP plus either 250 mM KCl (for KIF3AC) or 300 mM KCl (for KIF3AA and KIF3CC), 150 μM MEG, and 0.05 units/ml PNPase. For kinesins, the added salt is used to weaken rebinding of the kinesin head after the first ATP turnover and thereby slow subsequent turnovers of ATP so that the first and second turnovers can be better defined (24, 25).

The experiments presented in Fig. 2 were repeated five to eight times for KIF3AC and two times for KIF3AA and KIF3CC. Each transient is an average of 8 to 10 individual transients, and each transient represents fluorescence data for 1000 time points. To compare the fluorescence data for each MgATP concentration, the transients were adjusted by adding or subtracting an offset such that each transient began at the same point on the *y*-axis, arbitrary set at 1. This normalization approach was guided by the exponential fit to the transients to identify the point on the *y*-axis at time 0. The family of transients presented in Fig. 2 for each motor (KIF3AC, KIF3AA, and KIF3CC) are from experiments performed on the same day. The hyperbolic fit to the data in Fig. 2*B*, *D*, *F* provide the mean ± S.E.

### Mathematical modeling

A nonlinear ODE model was derived to simulate the time-dependent phosphate release fluorescence data. These ODEs represent the change in concentration of different model states (*i.e.,* kinesin intermediates within the model and nucleotide concentration). The model contained eight different ODEs and nine different parameters (Fig. 3). In our earlier work (21), both experimental data and simulations helped guide the initial assignment of starting values for the parameters within the model (Tables 1 and 2). After the initial assignment of parameter values, select parameters were estimated, whereas other parameters were set to rates reported previously.

An iterative gradient-based estimation approach was conducted where model parameter values were estimated for each iteration and the model was simulated at each iteration to compare the predicted model outputs to the experimental data. The objective function used for estimation was the MSE between the predicted and measured outputs, which needed to be minimized. The resulting nonlinear programming problem was solved with the MATLAB function *fmincon*, and the ode solver used for simulating the model was ode45. In converting the concentration of phosphate to fluorescence, a factor λ was multiplied by the concentration of released phosphate. Transients for the modeling were allocated for training and validation. Training transients were used for fitting the model to the data, and validation transients were used for testing whether the model could predict the data that were not used for training. Approximately 75% of the total number of transients modeled were used for training, and approximately 25% were used for model validation. Finally, the MSE was used to determine the goodness of fit of the model to the fluorescence data.

### Parameter estimation

The parameter estimation problem can be presented by the following function:

$$\min_{p}\underset{i}{\sum }\underset{k}{\sum }{\left(y_{ik}-{\hat{y}}_{ik}\right)}^{2}$$

In the parameter estimation problem, the terms $y_{ik}\;\text{and}\;{\hat{y}}_{ik}$ represent the model and experimental fluorescence, respectively, for the *i*th condition and *k*th time. Because the number of turnovers could not be distinguished in the model, the fluorescence data were modeled up to only 0.1 s (200 time points) to ensure there were at most two turnovers, one for each head of the heterodimer. The time domain for two turnovers was calculated using the steady-state rate of 21 s^−1^ for KIF3AC (1/21 s^−1^ or 0.0476 s/turnover or ∼0.1 s). The parameter vector *p* for the model consisted of the individual parameters *k*_1a_, *k*_1c_, *k*_−1a_, *k*_−1c,_ *k*_2a_, *k*_2c_, *k*_Ua_, *k*_Uc_, and λ. In addition, the optimization option for *fmincon* used was the interior-point algorithm. The parameters selected for estimation were *k*_−1a_, *k*_−1c_, *k*_Ua_, *k*_Uc_, and λ. The lower and upper bounds for parameter estimates were one-hundredth the starting value and 100 times the starting value, respectively, for parameters *k*_Ua_, *k*_Uc_, and λ. In estimating parameters *k*_−1a_ and *k*_−1c_, the lower and upper bounds were one-hundredth the starting value and infinity, respectively. CIs were only determined for estimated parameters and estimated using the parameter profile likelihood approach with a confidence of 95% as described previously (21, 42).

### Data availability

The raw data from the stopped-flow experiments (Figs. 2 and 4) will be provided on request (Susan P. Gilbert, Rensselaer Polytechnic Institute; email: sgilbert@rpi.edu).

## Conflict of interest

The authors declare that they have no conflicts of interest with the contents of this article.
